# Trauma, Attempted Suicide, and Morning Cortisol in a Community Sample of Adolescents

**DOI:** 10.1002/jts.20516

**Published:** 2010-04

**Authors:** Robert Young

**Affiliations:** MRC Social & Public Health Sciences UnitGlasgow

## Abstract

Individuals exposed to trauma or who have attempted suicide may show abnormal cortisol profiles; those exposed to significant trauma show reduced, while those who attempt suicide show increased cortisol output, although the evidence is inconsistent. This study explores the associations between morning cortisol, trauma, and suicide attempts or ideation among young people. In a community-based sample of 501 15-year-olds, using data from a *DSM-IV*-compatible interview on suicidal-behavior/ideation, trauma, and morning cortisol, we found no association between these factors and morning cortisol. A significant gender interaction was found for those threatened with a weapon—men showing a negative and women a positive association, suggesting that any cortisol/trauma association may be partially explained by coexisting behavioral problems and gender.

The hormone Cortisol is part of the hypothalamic–pituitary–adrenocortical axis (HPA-axis), a key biological system involved in the regulation of the stress response. Dysfunction of this system has been (inconsistently) linked with stress-related psychiatric conditions such as depression (McEwen, 2000) and posttraumatic stress disorder (PTSD) or trauma in general (Miller, Chen, & Zhou, 2007). The link between PTSD/trauma has been extensively investigated among adults, but less so in adolescents. Although elevated cortisol levels among youth with PTSD symptoms has been reported (Carrion et al., 2002), findings are inconsistent (Weems & Carrion, 2007).

A recent meta-analysis of PTSD and basal cortisol levels concluded there was no systematic difference between adults with PTSD and controls, but that individuals exposed to trauma, with or without PTSD symptoms, demonstrated a lower or "flattened" cortisol profile (Meewisse, Reitsma, De Vries, Gersons, & Olff, 2007). A complementary meta-analysis of stress and adult HPA- axis function found trauma was associated with (marginally) lower morning cortisol (Miller et al., 2007). In tandem, accumulating (but inconsistent) evidence from studies of adults and adolescents suggests individuals who have attempted suicide demonstrate abnormal cortisol profiles (van Heeringen, 2003), with studies reporting both elevated (Mathew et al., 2003) and attenuated (Lindqvist, Isaksson, Traskman-Bendz, & Brundin, 2008) cortisol levels, or alternatively reporting no difference (Bergman & Brismar, 1994; Dahl et al., 1991) compared to controls. Given the inconsistency of results and small effect sizes, this study investigates the association between basal (morning) cortisol and both trauma exposure and suicidal behavior or ideation.

## METHOD

Data for this study come from a subsample of 602 pupils from four schools, surveyed as part of a larger study of 22 schools (3,194 pupils). In addition to survey measures, pupils in the subsample completed a computerized, *Diagnostic and Statistical Manual of Mental Disorders, Fourth Edition*- (*DSM-IV*; American Psychiatric Association, 1994) compatible, audio version of the Diagnostic Interview Schedule for Children (DISC; Costello, Edelbrock, Dulcan, Kalas, & Klaric, 1984), the Voice-DISC (Shaffer, Fisher, Lucas, Dulcan, & Schwab-Stone, 2000; West, Sweeting, Der, Barton, & Lucas, 2003). We report the details and design of the larger study elsewhere (Kelly, Young, Sweeting, Fischer, & West, 2008). Briefly, participating schools were situated in predominantly urban areas in and around Glasgow city in the West of Scotland. All relevant ethics board, schools, parents, and pupil approval was granted. The subsample was not intended to be representative, but demographically is comparable with the larger, broadly representative sample (Kelly et al., 2008). Probabilistic weights to compensate for nonresponders have been derived, but their use made no substantive difference to results. All pupils in the final year of statutory schooling were invited to participate via parental opt-out consent forms. The survey component took place in school-based sessions during the first class (approximately 9:00–09:50 a.m.). During sessions, pupils completed a questionnaire and provided two salivary cortisol samples. The psychiatric component took place within a day of the standard survey. Five hundred sixty-two pupils (93% of the total subsample) completed a questionnaire and provided cortisol samples, of these, 501 pupils (89%) completed all relevant components of the voice-DISC. The mean age of this final subsample was 15.3 years.

The Voice-DISC is a replica of the interviewer version of the DISC and preliminary work suggests it is at least as reliable (Lucas, 2003). Respondents self-administer the DISC using a laptop computer. The PTSD section asks about exposure to nine common forms of trauma (e.g., natural disaster, see Table 1). Summated total trauma score and binary, disaster or accident, interpersonal trauma, witnessing/anticipating trauma, and any trauma variables were created. Only 11 participants experienced recent (last 4 weeks) trauma; therefore, we focused on lifetime exposure. The mood disorder section asked about past suicide attempts or ideation and recent thoughts about death. Nine participants were given a *DSM-IV* diagnosis for mood disorder and two for PTSD, neither diagnosis was associated with cortisol (Young, Sweeting, & West, 2009).

**Table 1:** Frequency of Trauma Exposure, Suicidal Thoughts and Behaviors, and Their Association With Morning Cortisol

	Exposure					Association with morning cortisol^a^					
		
	Male adolescent		Female adolescent			Male adolescent			Female adolescent		
					
Trauma type	*n*	%	*n*	%	χ^2^ test	*B*	*SE*	β	*B*	*SE*	β
Natural disaster	9	3.7	8	3.1	<1.00	†			†		
In bad accident	32	13.1	24	9.4	1.71	.012	.042	.017	.074	.047	.090
Any above, disaster or accident	36	14.7	27	10.5	1.96	.009	.040	.014	.084	.044	.108
Attacked or badly beaten	43	17.6	14	5.5	18.13***	.012	.037	.020	.105	.060	.100
Forced sexually	1	0.4	10	3.9	7.13**	†			†		
Threatened with weapon^b^	53	21.6	13	5.1	30.00***	−.058	.034	−.102	.110	.062	.101
Any above, interpersonal trauma	73	29.8	27	10.5	27.84***	−.032	.030	−.062	.066	.044	.085
‘Significant other’ at risk of harm	34	13.9	50	19.5	2.87	.019	.040	.027	−.019	.035	−.031
Witnessed death or severe injury	96	39.2	71	27.7	7.39**	−.006	.029	−.013	.020	.031	.036
Witnessed dead body	36	14.7	60	23.4	6.18*	−.025	.040	−.038	−.002	.032	−.004
Any above, witnessed/anticipated death or injury to others	121	49.4	117	45.7	<1.00	−.022	.028	−.047	.020	.027	.041
Any trauma	151	61.6	128	50.0	6.87**	−.002	.029	−.004	.045	.027	.095
Total trauma score^c^						−.007	.011	−.036	.013	.011	.071
Suicidal thoughts and behaviors											
Recent (past month) thoughts about death and dying	47	19.2	84	32.8	12.04***	.042	.036	.071	.014	.029	.028
Any suicidal ideation/attempts	4	1.6	24	9.4	14.22***	†			.037	.047	.045

*Note*.

† = regression analysis omitted due to small *n*.

a Adjusted for, time of awakening and time of collection. Each row reports a separate regression model.

b Significant gender interaction for morning cortisol levels, Δ *F* (1) = 5.69, *p* < .05. All other gender interactions are nonsignificant.

c Total trauma score for overall sample, *N* = 501, *M* = 1.11, *SD* = 1.29; male *n* = 245, *M* = 1.24, *SD* = 1.29; female *n* = 256, *M* = 0.98, *SD* = 1.28. Significant gender difference in total trauma score, *t*(499) = 2.30, *p* < .05.

* *p* < .05.

** *p* < .01.

*** *p* < .001.

Cortisol was obtained using a Salivette (Sarstedt AG & Co., Nümbrecht, Germany) sampling device 5 minutes into questionnaire completion using standard methodology (details available elsewhere; Kelly et al., 2008), with cortisol logged for analysis. Because results were similar for both time points, only those for Time 1 (T1) are reported. Sampling at 9:00 a.m. was chosen to avoid the cortisol-awakening response and because morning cortisol levels are considerably more reliable (Kirschbaum et al., 1990). We did not average our measures to avoid introducing potential complications in interpretation, e.g., different associations with cortisol may be found at different times of the day (Hruschka, Kohrt, & Worthman, 2005), and because results using the average were virtually identical to those for T1. Here we adjusted for potential confounds both related to the biology of circadian rhythm and found to be significant in our previous article (Kelly et al.), these being awakening time and time of first sample.

Analysis used a series of separate regressions with morning cortisol predicted by either trauma or suicide-related variables, adjusted for cortisol confounds and analyzed separately by gender. One multivariate outlier was identified and excluded. Tests for gender interactions were conducted. Gender differences in trauma exposure were explored using the chi-square test. Power calculations suggest we have over 80% power to detect a small association (*N* = 501, *r* = .13, *p* < .05, two-sided).

## RESULTS

Exposure (*n* and %) to each form of trauma is shown in Table 1, as are the rates of suicide attempt/ideation or thoughts about death. Although not intended to provide a prevalence estimate and acknowledging differences in age and context, our rates are broadly compatible with retrospective accounts of childhood trauma exposure (Briere, Kaltman, & Green, 2008). There were significant gender differences in exposure, with male adolescents exposed to greater violence-related (e.g., attacked or badly beaten) and overall trauma, and female adolescents exposed to greater sexually related trauma (forced sexually) and more likely to witness a dead body. Other specific types of trauma were unrelated to gender. All suicide-related outcomes were more frequent among female adolescents. No association between cortisol and trauma exposure, suicide attempt/ideation, or thoughts about death was significant (see Table 1). Among female adolescents, morning cortisol was higher in those exposed to any “accident or disaster” and those attacked or badly beaten, threatened with weapon, or exposed to any trauma, but these were small effect-sizes of marginal significance (β ≈ .10). Among male adolescents, being threatened with a weapon was marginally associated with lower cortisol, with a formal test demonstrating a significant gender interaction (*p* < .05).

## DISCUSSION

Despite finding that exposure to different forms of trauma, attempted suicide, and ideation varied considerably by gender, contrary to expectations we found no evidence that these were related to morning cortisol levels. Exposure to different forms of trauma patterned according to gender, i.e., female adolescents were exposed to more sexually related and male adolescents more violence-related trauma. Our findings confirm that exposure to various forms of trauma appears relatively common during childhood and adolescence (Briere et al., 2008; American Academy of Child & Adolescent Psychiatry, 1998).

Our results suggest a lack of association between exposure to trauma and basal cortisol levels; however, there are at least two additional factors to consider. First, some individuals may be more vulnerable to exposure to certain types of trauma. Previously, we found several traumatic and life events not specifically assessed by the PTSD module were associated with elevated cortisol, notably, recent death of a friend, or new step-parent (Kelly et al., 2008).The design of this study cannot address all potential biopsychosocial mechanisms linked with vulnerability to stress, including the growing evidence of gene-environment interactions related to cortisol functioning (Tyrka et al., 2009). We found completely opposite patterns of association between cortisol and being threatened with a weapon for each gender. This could be explained by gender differences in the association between cortisol and conduct disorder. In a related article using the same sample, we found that conduct symptoms are positively associated with cortisol for female adolescents, but negatively for male adolescents (Young et al., 2009). There is limited evidence to the suggestion that female adolescents exposed to accidents or disaster or any trauma show elevated cortisol, compatible with some of the literature (Carrion et al., 2002), but formal tests for gender differences were nonsignificant.

Second, the degree of trauma experienced by participants could be of greater severity or duration than is assessed by the Voice-DISC. Potentially, different results may have been obtained either by the use of more extensive measures; from adult, children, or clinical samples; from those exposed to recent or more severe trauma (Weems & Carrion, 2007); or those who recently attempted suicide. One hypothesis suggests that recent trauma is positively, but distal trauma is negatively, associated with cortisol (Weems & Carrion, 2007); however, because the Voice-DISC only distinguishes between lifetime and recent (past month) trauma it impossible to test this. Notwithstanding such caveats, our findings are compatible with other recent population studies (Sondeijker et al., 2007) and suggest a need to critically evaluate empirical evidence linking cortisol and PTSD, trauma, and suicide-risk among adolescents.
